# Data and Statistical Methods To Analyze the Human Microbiome

**DOI:** 10.1128/mSystems.00194-17

**Published:** 2018-03-13

**Authors:** Levi Waldron

**Affiliations:** a Graduate School of Public Health and Health Policy, City University of New York, New York, New York, USA; b Institute for Implementation Science in Population Health, City University of New York, New York, New York USA

**Keywords:** machine learning, meta-analysis, metagenomics, statistical analysis

## Abstract

The Waldron lab for computational biostatistics bridges the areas of cancer genomics and microbiome studies for public health, developing methods to exploit publicly available data resources and to integrate -omics studies.

## PERSPECTIVE

The rapidly developing field of human microbiome studies will benefit from adapting the statistical and computational methods of more mature areas of high-dimensional data analysis and from ongoing use of the growing catalog of publicly available microbiome data. This perspective discusses methods and resources for robust identification of differentially abundant microbes and predictive models of microbiome-linked health outcomes. I summarize lessons from high-dimensional data analysis for cancer genomics and efforts by my lab to leverage and adapt the Bioconductor project for analysis and comprehension of high-throughput genomic data (1) to bring value-added published data, meta-analysis, and methods for multiomic data analysis to the microbiome community.

## COMPARATIVE ANALYSIS AND META-ANALYSIS FOR DIFFERENTIAL ABUNDANCE

Differential abundance analysis is probably the most common objective of microbiome profiling studies and genomics studies in general. The objective is to identify microbial taxa, anywhere on the tree of life, that are over- or underabundant in some condition relative to a reference condition. These conditions can be observed or experimentally determined. The most commonly used methods for differential abundance analysis are LEfSe (2) and a variety of tools based on log linear regression models with negative binomial (3) or zero-inflated Gaussian error models (4). Regression approaches involve a false-discovery rate estimation to correct for multiple-hypothesis testing. Log linear modeling approaches build on a large body of statistical and computational work and provide several practical advantages. First, regression approaches eliminate the need for rarefaction, a process that has been described as “inadmissable” for the identification of differentially abundant taxa (5) because it throws away potentially useful data, the extra reads from samples with greater sequencing depth. Second, they adapt empirical Bayesian methods developed to reduce false-positive results in microarray differential expression analysis by “borrowing” information across taxa on how taxa are distributed across samples. Finally, they accommodate multivariate models that can be used for causal inference, such as to control for confounding effects or to test hypotheses of the microbiome as a mediator between environmental exposure and health outcomes. Regression modeling, now the almost exclusive choice for differential expression analysis of RNA sequencing data, is also well suited to metatranscriptomic differential abundance analysis.

These efforts can be enhanced by the standardization and reuse of published data for meta-analysis, comparative analysis, and method development. Thus, my lab developed the curatedMetagenomicData database (6) in collaboration with the laboratories of Nicola Segata (MetaPhlAn2 [7] and other methods for metagenomics), Curtis Huttenhower (developers of the bioBakery [8] and many methods therein), and Martin Morgan (head of the Bioconductor project [1]). This database provides more than 6,000 human-associated shotgun metagenomic profiles, uniformly processed from raw sequencing data to provide taxonomic abundance (7) and metabolic functional potential (9). Samples are primarily from stool specimens but include the Human Microbiome Project and other data sets sampling from other human body sites. We developed a fully automated, cloud-based pipeline to facilitate ongoing addition and updating of the database as new metagenomes and reference genomes become available and to encourage community contributions and even creation of alternative and competing databases.

## MULTIOMIC INVESTIGATION OF THE MICROBIOME

Metagenomic studies, as in other areas of genomics, increasingly incorporate multiple assays in an experiment. My lab recently published MultiAssayExperiment (10), software for the integration of multiomics experiments in Bioconductor. MultiAssayExperiment has enabled coordinated representation and manipulation of multiple -omics data types for 11,000 patients and 33 cancers studied as part of the Cancer Genome Atlas. A more complete picture of host-microbiome relationships may also be developed by collecting multiple -omics data types, and I have been involved in studies including metatranscriptomics (11) and host gene expression (12) in addition to taxonomic and functional microbiome abundance data. To overcome the complexity of reproducible data analysis and interpretation of such experiments, I am working with other Bioconductor microbiome package developers to create a common standard for representing microbiome data. This standard will provide compatibility with MultiAssayExperiment and with recent advances based on HDF5 and Google BigTable for on-disk data and remote representation of very large data. This will, for example, allow curatedMetagenomicData (6) to represent taxonomic, gene family, and metabolic functional profiles for more than 6,000 samples as a single Bioconductor object that users can interact with in almost the same way as they currently do with microbiome (4, 13) or gene expression data from a single study, even on a standard laptop.

## PREDICTIVE MODELING/MACHINE LEARNING

Prediction of health outcomes is a complementary objective to differential abundance analysis. Although similar models are sometimes used for these different objectives, the objective of making accurate predictions motivates different methods for model development and assessment. A mainstream approach to prediction modeling in high-dimensional data is to apply multivariate penalized regression, or machine learning methods such as Support Vector Machine, in conjunction with cross-validation to assess prediction accuracy. These approaches have been quickly adopted for prediction of health status from microbiome data. Colleagues and I have previously shown in meta-analyses of cancer transcriptomes that such approaches are prone to overoptimistic estimation of prediction accuracy (14). There are numerous possible reasons for such overoptimism. The data used to develop prediction models are by necessity retrospective, meaning they are predicting the past and not the future. “Information leakage” in data set through incorrect cross-validation, “reverse causality” effects of treatment on the microbiome, batch effects introduced by knowledge of outcomes, for example by sequencing cases together and then sequencing controls in another batch. Most studies do not collect statistically random samples, and therefore, the samples are not representative of the population.

Even with these challenges, it is sometimes still possible to develop accurate models of disease state and outcome from high-dimensional data. Colleagues and I showed that systematic leave-one-data set-in cross-study validation (15) of independent publicly available data sets provides a more realistic picture of generalizable prediction accuracy and that heterogeneous studies can be used to train robust prediction models through leave-one-data set-out cross-study validation (16). We have also shown the value of these approaches for metagenomic prediction problems (17). In systematic cross-study validation of gene expression-based models of cancer patient prognosis, we have shown even simple and suboptimal machine learning algorithms to be competitive with complex, theoretically optimal methods (18). Standardized databases like curatedMetagenomicData (6) and our in-development HMP16SData package (http://bioconductor.org/packages/HMP16SData/) will facilitate future work to find the limits of accuracy for disease prediction from all available microbiome profiles.

## FUTURE OUTLOOK

Discoveries that are replicable across independent experiments are more likely to be valid and useful than those seen only in a single data set. My research aims to harness publicly available microbiome data through curation, integration and standardization, novel reanalysis, and methodological development. I aim to ensure that studies of the human microbiome benefit from concurrent methodological development in other areas of genomics and from the growing body of publicly available microbiome data. These benefits include more reliable identification of differentially abundant microbial species, strains, and community structure and the development of disease prediction models that hold up to independent validation across populations. I see the Bioconductor project as providing a unique opportunity for the microbiome community to leverage more than 15 years of development of statistical methods for -omics data and to integrate microbiome data with other types of high-throughput data. As such, I plan to continue developing the Bioconductor platform to the needs of the microbiome community, through the development of databases, promotion of standards for data representation, and development of needed methods for data manipulation and analysis.
